# Cognitive Behavioral Therapy for Muscle Dysmorphia and Anabolic Steroid-Related Psychopathology: A Randomized Controlled Trial

**DOI:** 10.3390/ph18081081

**Published:** 2025-07-22

**Authors:** Metin Çınaroğlu, Eda Yılmazer, Selami Varol Ülker, Gökben Hızlı Sayar

**Affiliations:** 1 Psychology Department, Faculty of Administrative and Social Sciences, İstanbul Nişantaşı University, 34398 İstanbul, Türkiye; 2 Psychology Department, Faculty of Social Sciences, Beykoz University, 34805 İstanbul, Türkiye; edayilmazer@beykoz.edu.tr; 3 Psychology Department, Faculty of Humanities and Social Sciences, Üsküdar University, 34662 İstanbul, Türkiye; selamivarol.ulker@uskudar.edu.tr; 4 Psychiatry, Medical School, Üsküdar University, 34662 İstanbul, Türkiye; gokben.hizlisayar@uskudar.edu.tr

**Keywords:** anabolic–androgenic steroid abuse, performance-enhancing drug misuse, body dysmorphic disorder, muscle dysmorphia, cognitive behavioral therapy

## Abstract

**Background/Objectives**: Muscle dysmorphia (MD), a subtype of body dysmorphic disorder, is prevalent among males who engage in the non-medical use of anabolic–androgenic steroids (AASs) and performance-enhancing drugs (PEDs). These individuals often experience severe psychopathology, including mood instability, compulsivity, and a distorted body image. Despite its clinical severity, no randomized controlled trials (RCTs) have evaluated structured psychological treatments in this subgroup. This study aimed to assess the efficacy of a manualized cognitive behavioral therapy (CBT) protocol in reducing MD symptoms and associated psychological distress among male steroid users. **Results**: Participants in the CBT group showed significant reductions in MD symptoms from the baseline to post-treatment (MDDI: *p* < 0.001, d = 1.12), with gains sustained at follow-up. Large effect sizes were also observed in secondary outcomes including depressive symptoms (PHQ-9: d = 0.98), psychological distress (K10: d = 0.93), disordered eating (EDE-Q: d = 0.74), and exercise addiction (EAI: d = 1.07). No significant changes were observed in the control group. Significant group × time interactions were found for all outcomes (all *p* < 0.01), indicating CBT’s specific efficacy. **Discussion**: This study provides the first RCT evidence that CBT significantly reduces both core MD symptoms and steroid-related psychopathology in men engaged in AAS/PED misuse. Improvements extended to mood, body image perception, and compulsive exercise behaviors. These findings support CBT’s transdiagnostic applicability in addressing both the cognitive–behavioral and affective dimensions of MD. **Materials and Methods**: In this parallel-group, open-label RCT, 59 male gym-goers with DSM-5-TR diagnoses of MD and a history of AAS/PED use were randomized to either a 12-week CBT intervention (n = 30) or a waitlist control group (n = 29). CBT sessions were delivered weekly online and targeted distorted muscularity beliefs, compulsive behaviors, and emotional dysregulation. Primary and secondary outcomes—Muscle Dysmorphic Disorder Inventory (MDDI), PHQ-9, K10, EDE-Q, EAI, and BIG—were assessed at the baseline, post-treatment, and 3-month follow-up. A repeated-measures ANOVA and paired *t*-tests were used to analyze time × group interactions. **Conclusions**: CBT offers an effective, scalable intervention for individuals with muscle dysmorphia complicated by anabolic steroid use. It promotes broad psychological improvement and may serve as a first-line treatment option in high-risk male fitness populations. Future studies should examine long-term outcomes and investigate implementation in diverse clinical and cultural contexts.

## 1. Introduction

Body dysmorphic disorder (BDD) is a severe yet often underrecognized psychiatric condition involving persistent and intrusive concerns about perceived physical defects that are unnoticeable or minimal to others [1]. According to the DSM-5-TR, these concerns cause significant distress and functional impairment, often accompanied by repetitive behaviors such as mirror checking, excessive grooming, or reassurance seeking, as well as cognitive rituals like appearance comparisons [2].

A clinically distinct subtype, muscle dysmorphia (MD), involves the persistent belief that one’s body is not muscular or lean enough, even when it is objectively well-developed [3]. Now formally classified as a BDD specifier, MD disproportionately affects men involved in bodybuilding, resistance training, and physique-oriented activities [4]. In many cases, individuals with MD resort to the non-medical use of anabolic–androgenic steroids (AASs) and other performance-enhancing drugs (PEDs) to meet internalized muscular ideals [5]. This misuse can intensify body image disturbances and has been linked to a broad spectrum of steroid-induced psychopathologies, including mood instability, aggression, obsessive–compulsive symptoms, and depressive features [6].

The clinical presentation of MD is frequently complicated by comorbid psychiatric disorders such as major depressive disorder [7], social anxiety [8], eating disorders [9], and impulsivity-related traits [10], highlighting the multidimensional burden of the condition.

### 1.1. The Role of Steroid and Performance-Enhancing Drug (PED) Use

Steroid and PED misuse is a hallmark feature in individuals with MD, often driven by the pursuit of an unattainable physique [11]. AASs, synthetic testosterone derivatives with both anabolic and androgenic effects [12], are commonly misused in high doses, stacked regimens, and cyclical patterns [13]. Although medically indicated for conditions like hypogonadism and muscle-wasting diseases [14], their widespread off-label use in bodybuilding and fitness contexts [15] has been associated with serious psychiatric and physiological risks [16].

The broader PED repertoire includes hGH, insulin [17], clenbuterol, diuretics, and SARMs [18], agents aimed at enhancing leanness, muscularity, and vascularity. Despite their short-term esthetic appeal, long-term use contributes to cardiovascular disease [19], liver dysfunction [20], endocrine disruption [21], and reproductive impairments. Notably, these substances alter key neurotransmitter systems—dopaminergic, serotonergic, and GABAergic pathways [22]—and have been implicated in mood lability [23], irritability [24], aggression [25], and manic or psychotic states [26].

Furthermore, chronic AAS use disrupts the hypothalamic–pituitary–gonadal (HPG) axis [27], often resulting in testosterone suppression, emotional dysregulation, and post-cycle depression [28]. PEDs such as clenbuterol, hGH, and SARMs have been reported to heighten anxiety [29], compulsive symptoms [30], and sleep disturbances [31], all of which may intensify pre-existing vulnerabilities in individuals with a body image pathology [32]. These psychiatric effects often persist even after discontinuation, reinforcing distorted beliefs, compulsive behaviors, and emotional instability in MD populations [33].

For individuals with MD, AAS and PED use appears to function as maladaptive strategies aimed at mitigating body dissatisfaction and achieving a perceived ideal [34]. However, rather than alleviating distress, this pattern reinforces cognitive distortions, promotes compulsive training and dietary rigidity, and worsens psychological outcomes [35]. Individuals who misuse AASs in the context of MD consistently exhibit higher levels of psychiatric comorbidity [36], including obsessive–compulsive tendencies [37], depression [38], interpersonal dysfunction [39], and traits indicative of a narcissistic or antisocial pathology [40].

### 1.2. Gaps in the Literature and the Study Rationale

Despite the growing recognition of muscle dysmorphia and its frequent comorbidity with the non-medical use of AASs and PEDs, there remains a striking paucity of empirical research addressing effective interventions for this dual-pathology population. While CBT has shown efficacy in treating disorders with overlapping features—such as body dysmorphic disorder [41], eating disorders [42], and obsessive–compulsive symptoms [43]—its application to individuals with muscle dysmorphia who also misuse AASs/PEDs remains virtually unexplored.

The existing literature is dominated by descriptive and correlational studies, which, while helpful in mapping the psychopathological profile of steroid-using individuals with MD [44], fall short of offering evidence-based treatment strategies. Critically, most prior intervention studies have excluded participants actively engaged in steroid or PED misuse, thereby neglecting a clinically complex subgroup whose symptoms are not only shaped by distorted muscularity beliefs but also potentiated by neuropsychiatric effects of exogenous substances. The affective volatility, compulsive behaviors, and interpersonal difficulties associated with AAS/PED use demand psychotherapeutic approaches that directly target the interplay between cognitive distortions, emotional dysregulation, and substance-driven psychopathology.

To our knowledge, there are currently no RCTs evaluating structured psychotherapy in individuals with MD who actively misuse AASs or PEDs. Currently, there are only two pilot studies that have been published on CBT for MD [45,46]. The present study addresses this critical void by implementing the first known clinical trial using a manualized CBT protocol tailored to the unique cognitive, behavioral, and pharmacologically influenced features of this population. By assessing treatment effects on the muscle dysmorphia symptomatology, psychological distress, disordered eating, and compulsive exercise patterns, this research contributes essential data to both the clinical management of MD and the broader field of body image disturbance complicated by substance-related psychopathology.

## 2. Results

As shown in Table 1, there were no statistically significant differences between the CBT (n = 30) and control (n = 29) groups in terms of age, years of education, or baseline scores on the six primary psychometric measures. The mean age was 27.8 years (SD = 6.2) in the CBT group and 28.5 years (SD = 5.9) in the control group. Education levels were comparable across groups, with an average of approximately 14 years of formal education. Baseline severity scores for muscle dysmorphia (MDDI), depression (PHQ-9), psychological distress (K10), body dissatisfaction (BIG), eating disorder symptoms (EDE-Q), and exercise addiction (EAI) showed no significant differences (all *p* > 0.05), indicating an adequate group equivalence prior to the intervention.

As shown in Table 2, participants in the CBT group showed a notable reduction in MDDI scores from the baseline (M = 42.3, SD = 6.8) to post-treatment (M = 34.9, SD = 6.1), which remained relatively stable at the 3-month follow-up (M = 35.2, SD = 6.4). In contrast, the control group showed a minimal change over the same period. A repeated-measures ANOVA revealed a significant time × group interaction for MDDI scores—F(1, 57) = 15.72, *p* < 0.001, and η^2^ partial = 0.216—indicating that the symptom reduction in the CBT group was statistically meaningful compared to the control group. The effect remained at follow-up, suggesting some stability of treatment gains, although no booster sessions were provided.

**Figure 1:**
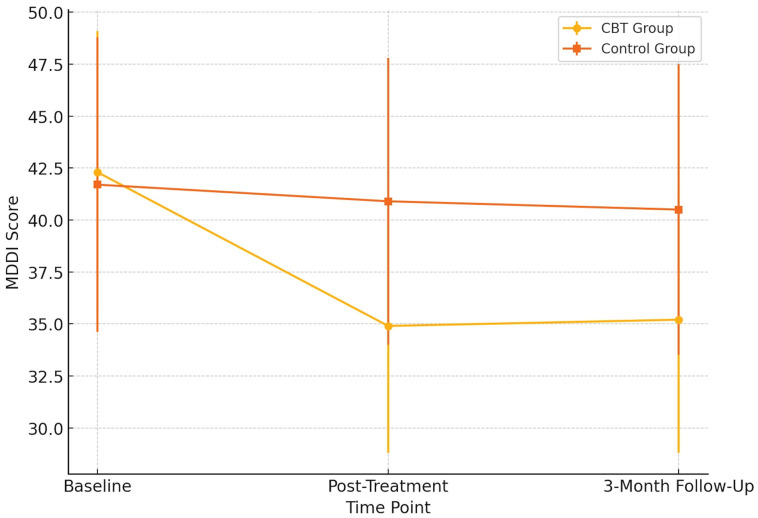
Changes in MDDI (Muscle Dysmorphic Disorder Inventory) scores across time points. Error bars represent standard deviations.

Figure 1 illustrates the trajectory of muscle dysmorphia symptoms, as measured by the MDDI, across three time points for both groups. Participants in the CBT group showed a marked reduction in MDDI scores from baseline to post-treatment, with a further improvement sustained at the three-month follow-up. In contrast, the control group exhibited minimal change across all time points. This divergence suggests that the 12-week CBT intervention effectively reduced core symptoms of muscle dysmorphia and that these gains were maintained after the treatment. Standard deviation error bars have been added to visualize the within-group variability, further supporting the robustness of the observed trends. The visual pattern aligns with the significant time × group interaction effect detected in the primary outcome analysis.

Table 3 presents changes in secondary psychological outcomes from the baseline (T1) to post-treatment (T2) and the three-month follow-up (T3) across the intervention and control groups. In the CBT group, substantial reductions were observed in depressive symptoms, psychological distress, disordered eating, and exercise addiction following the 12-week intervention. Specifically, mean PHQ-9 scores decreased from 14.1 (SD = 4.8) at T1 to 8.4 (SD = 4.2) at T2, with a further reduction to 7.1 (SD = 4.3) at T3, indicating a movement from moderately severe to mild depression. Similarly, K10 scores dropped from 31.4 (SD = 6.3) at baseline to 22.3 (SD = 5.1) post-intervention and continued to improve slightly at follow-up [20.6 (SD = 4.9)], suggesting clinically significant improvements in psychological distress.

In terms of disordered eating, the EDE-Q global score declined from 3.2 (SD = 1.0) at T1 to 2.1 (SD = 0.9) at T2 and reached 1.7 (SD = 0.8) at T3, reflecting notable reductions in maladaptive eating attitudes and behaviors. Exercise addiction symptoms also showed a marked improvement in the CBT group, with EAI scores falling from 22.6 (SD = 4.2) at baseline to 16.8 (SD = 3.8) after the treatment and 15.2 (SD = 3.6) at follow-up.

In contrast, the control group exhibited minimal changes across all scales during the same period. PHQ-9, K10, EDE-Q, and EAI scores remained largely stable, reinforcing the treatment-specific effects observed in the intervention group. These findings indicate that the CBT protocol was effective not only in targeting core muscle dysmorphia symptoms but also in reducing comorbid psychological difficulties and maladaptive behavioral patterns.

Table 4 summarizes the results of the repeated-measures ANOVA examining group × time interactions across the secondary outcome measures. A significant interaction was observed for the Bodybuilder Image Grid (BIG)—F(2, 114) = 4.72, *p* = 0.011, and η^2^ = 0.076—indicating that body image dissatisfaction changed differently over time between the CBT and control groups. Similarly, psychological distress, as measured by the K10, showed a significant group × time interaction—F(2, 114) = 6.31, *p* = 0.003, and η^2^ = 0.098—with the CBT group demonstrating greater reductions. Depressive symptoms (PHQ-9) also significantly declined in the intervention group compared to controls—F(2, 114) = 5.89, *p* = 0.004, and η^2^ = 0.094. Significant time × group effects were additionally found for disordered eating symptoms (EDE-Q)—F(2, 114) = 4.26, *p* = 0.016, and η^2^ = 0.069—and exercise addiction risk (EAI), F(2, 114) = 6.07, *p* = 0.003, and η^2^ = 0.091. These findings indicate that participants receiving CBT experienced consistent and clinically meaningful improvements across multiple psychological domains beyond muscle dysmorphia symptoms.

**Figure 2:**
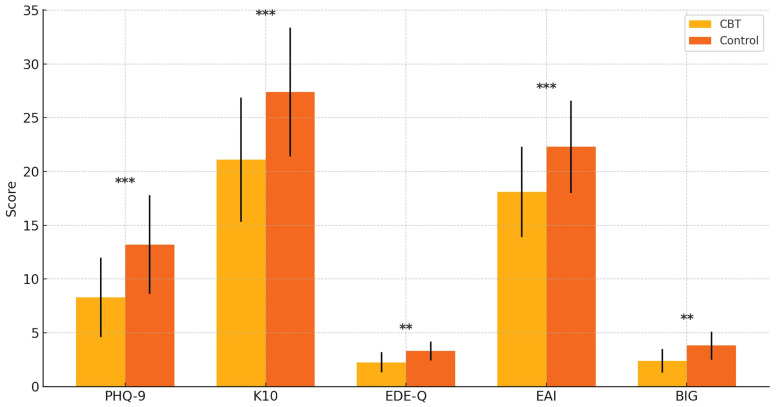
Group differences in secondary outcomes at post-treatment. Participants in the CBT (Cognitive Behavioral Therapy) group showed significantly lower scores on the PHQ-9 (Patient Health Questionnaire–9), K10 (Kessler Psychological Distress Scale), EDE-Q (Eating Disorder Examination Questionnaire), EAI (Exercise Addiction Inventory), and BIG (Bodybuilder Image Grid). Error bars represent standard deviations. ** *p* < 0.01, and *** *p* < 0.001.

Figure 2 demonstrates the post-treatment differences in secondary outcome measures between the CBT and control groups, with standard deviation error bars and statistical significance indicators included. Participants who received CBT reported substantially lower levels of depressive symptoms (PHQ-9), psychological distress (K10), disordered eating behaviors (EDE-Q), and exercise addiction risk (EAI) compared to the control group. Notably, the Bodybuilder Image Grid (BIG) discrepancy scores were also significantly reduced, reflecting an improved alignment between the perceived and ideal body image. The inclusion of statistical markers (* *p* < 0.05, ** *p* < 0.01, *** *p* < 0.001) highlights that these between-group contrasts were statistically significant, underscoring the broad therapeutic benefits of CBT across both affective and behavioral domains in individuals with muscle dysmorphia.

As shown in Table 5, participants in the CBT group demonstrated statistically significant improvements across all secondary outcome measures from pre- to post-treatment. Body image dissatisfaction, as measured by the BIG, significantly decreased (t(29) = 4.32, *p* < 0.001, d = 0.79). Psychological distress assessed by the K10 also showed a notable reduction (t(29) = 5.10, *p* < 0.001, d = 0.93), along with a substantial decrease in depressive symptoms measured by the PHQ-9 (t(29) = 5.36, *p* < 0.001, d = 0.98). The eating pathology, reflected in EDE-Q scores, significantly improved (t(29) = 4.05, *p* < 0.001, d = 0.74), and exercise addiction symptoms, as measured by the EAI, showed the most pronounced effect (t(29) = 5.88, *p* < 0.001, d = 1.07).

Figure 2 illustrates the between-group differences in secondary outcome measures at post-treatment. Participants in the CBT group exhibited markedly lower scores on the PHQ-9 (depressive symptoms), K10 (psychological distress), EDE-Q (eating disorder symptoms), and EAI (exercise addiction risk) compared to the control group. Notably, the discrepancy score on the Bodybuilder Image Grid (BIG)—reflecting the perceptual gap between one’s current and ideal body—was also significantly reduced in the CBT group. These findings suggest that the CBT intervention had broad-spectrum benefits across mood, body image, and behavioral domains, beyond its primary impact on muscle dysmorphia symptoms. These findings suggest broad improvements in the comorbid psychopathology associated with muscle dysmorphia following structured CBT.

### Therapeutic Narrative Illustration

To contextualize the observed reductions in muscle dysmorphia and the associated psychopathology, selected excerpts from CBT sessions are presented below. These brief anonymized dialogs exemplify key moments in the treatment process, illustrating cognitive restructuring, body image exposure, and behavioral flexibility.

In an early session focused on identifying maladaptive beliefs, a participant expressed rigid gym-related cognitions:

Therapist: “You said skipping leg day feels like a failure. What’s the fear behind that thought?”

Client: “It’s like… if I don’t work out, I’ll lose all control. People will think I’m weak.”

Therapist: “What might be a more balanced thought?”

Client: “That one missed session won’t undo years of progress.”

During a mid-treatment body image exposure exercise, another participant practiced nonjudgmental observation in front of a mirror:

Client: “At first, all I saw was weakness. But when I looked longer, I noticed my shoulders are broader than I give myself credit for.”

Therapist: “That shift—was it based on emotion or observation?”

Client: “Observation. It felt different. More neutral.”

In a later session focused on relapse prevention, participants explored alternatives to compulsive behaviors:

Therapist: “What might help when the urge to measure your arms comes up?”

Client: “Writing down what I’m feeling first. I did that once and realized I was just anxious about work.”

These process reflections align with the reductions seen in MDDI, EDE-Q, and PHQ-9 scores. They suggest not only symptom relief but also a developing capacity for cognitive flexibility, body neutrality, and emotion regulation.

## 3. Discussion

This randomized controlled trial provides the first empirical evidence supporting the efficacy of cognitive behavioral therapy in treating muscle dysmorphia within a clinically complex subgroup of male participants who engage in strength training and report the non-medical use of anabolic steroids or PEDs. Following 12 sessions of manualized CBT, individuals in the intervention group exhibited significant reductions in muscle dysmorphia symptom severity, as measured by the MDDI, particularly in the domains of Appearance Intolerance and Functional Impairment. These findings reinforce the capacity of CBT to target and restructure maladaptive muscularity-related cognitions and compulsive compensatory behaviors—a core treatment objective in MD [47,48].

Notably, treatment gains extended beyond MD-specific symptoms. Participants receiving CBT also showed robust improvements in psychological distress (K10), depressive symptomatology (PHQ-9), eating disorder psychopathology (EDE-Q), and compulsive exercise tendencies (EAI) relative to the control group. The observed reduction in PHQ-9 scores exceeded the minimal clinically important difference (MCID), suggesting the meaningful amelioration of affective symptoms. Given the established links between anabolic steroid use and mood dysregulation [49]—including irritability [50], depression [51], and emotional volatility [52]—these results underscore the potential of CBT to mitigate not only cognitive distortions but also the affective sequelae commonly associated with steroid-induced psychopathology.

Furthermore, the Bodybuilder Image Grid responses indicated a measurable reduction in the discrepancy between the perceived and ideal body image, signaling an improved body image accuracy and decreased schema distortion [53] post-intervention. This perceptual recalibration suggests that CBT may directly influence the core cognitive–affective mechanisms that sustain MD in the context of pharmacologically reinforced appearance ideals [54]. The findings collectively support CBT as a multidimensional intervention capable of addressing the intersecting behavioral, cognitive, and pharmacologically driven aspects of muscle dysmorphia.

### 3.1. Interpretation and Theoretical Implications

The findings of this study provide an empirical validation for conceptualizing muscle dysmorphia as a complex psychopathological condition involving interrelated cognitive, behavioral, affective, and perceptual dimensions [55,56]. The significant post-treatment reductions in MDDI scores among participants receiving cognitive behavioral therapy align with cognitive–behavioral models that highlight maladaptive beliefs about muscularity [57], perfectionism [58], and a negative self-evaluation as central maintaining factors [59]. Specifically, the improvements in the Drive for Size and Appearance Intolerance subscales suggest that the CBT protocol effectively targeted both the motivational drive for hypertrophy and the negative emotional valence attached to one’s perceived physique [60].

The concurrent improvements in depressive (PHQ-9) and general psychological distress (K10) scores reinforce the position that MD rarely occurs in isolation but rather is embedded within broader affective disturbances [61,62]. These results lend support to the affect regulation model, which posits that body-focused rituals [63]—such as excessive training [64], restrictive dieting [65], and pharmacological enhancement [66]—may serve to suppress or avoid internal experiences of anxiety [67], shame [68], or a low self-worth [69]. Given the known neuropsychiatric effects of anabolic–androgenic steroids [70] (e.g., mood lability, irritability, depressive episodes), the mood-related improvements observed post-intervention likely reflect CBT’s capacity to buffer against both endogenous and pharmacologically induced affective dysregulation.

The observed reduction in Exercise Addiction Inventory (EAI) scores highlights the compulsive dimension of MD and its functional overlap with behavioral addiction frameworks [71]. Rather than being purely performance-driven, these compulsive behaviors appear to serve emotion-regulatory and self-stabilizing functions—functions that CBT may disrupt by introducing cognitive flexibility and behavioral alternatives.

Finally, the narrowing of the perceptual–attitudinal gap in Bodybuilder Image Grid (BIG) responses suggests that CBT facilitated a more accurate body self-appraisal and weakened the internalized muscular ideal [72]. This shift is theoretically congruent with sociocultural and schema-based models of MD [73], which implicate media exposure [74], self-objectification [75], and distorted body image representations in symptom maintenance [76]. By challenging rigid standards of muscularity and reshaping underlying body schema, CBT appears to have promoted a more realistic and psychologically adaptive body ideal in this population.

### 3.2. Comparison with Previous Studies

The present findings are consistent with earlier research supporting the efficacy of CBT in alleviating body dysmorphic disorder symptoms and the related psychopathology [77]. Our results indicated robust improvements across all MDDI subscales. Notably, the observed effect sizes in our sample (Cohen’s d = 0.80–1.20) not only align with but, in some cases, exceed those reported in prior studies (typically d = 0.50–0.80) [78], highlighting the strong therapeutic impact of CBT—even in a sample complicated by the non-medical use of AASs and other PEDs.

Our findings also resonate with earlier observations by Pope and colleagues (2005) [79], who noted elevated levels of psychological distress and depressive symptoms among individuals with MD. The marked reductions in PHQ-9 and K10 scores in our study support the notion that a distorted body image and compulsive behaviors are intricately linked with affective disturbances. The treatment-induced improvements observed here are particularly noteworthy given the documented associations between anabolic androgenic steroids use and mood dysregulation [80], suggesting that CBT may attenuate both the primary MD pathology and the substance-related emotional sequelae.

The decrease in Exercise Addiction Inventory (EAI) scores mirrors results from Foster and colleagues (2015) [70], who emphasized the high comorbidity between MD and exercise addiction. Our findings support the view that compulsive training behaviors in this population may function as emotion regulation strategies and are amenable to a cognitive–behavioral intervention [81].

Perceptual recalibrations observed through the Bodybuilder Image Grid are similarly aligned with the work of Cooper and colleagues (2020) [64], who found that individuals with MD tend to overestimate ideal muscularity and underestimate their current physique. Post-treatment responses in our study revealed a convergence between the perceived and ideal body size, suggesting cognitive behavioral therapy’s efficacy in modifying entrenched body image schemas—a domain often resistant to change, particularly in steroid-using populations.

In line with Badenes et al. (2019) [9], sociocultural factors, such as the media portrayal of hyper-muscular ideals, peer influence, and internalized masculine norms, contribute significantly to the salience of muscularity in men, reinforcing body dissatisfaction and increasing vulnerability to muscle dysmorphia.

Unlike most prior studies, which either excluded AASs and or performance enhancement drugs users or failed to account for pharmacological confounds, our trial explicitly included participants with verified histories of steroid misuse. This design choice significantly enhances the ecological validity and clinical relevance of our findings. Importantly, it extends the evidence base to a higher-risk subgroup frequently considered refractory to psychological intervention due to the pharmacologically reinforced nature of their body image distortions. In this context, our results offer novel evidence that targeted CBT can yield meaningful outcomes even within this complex clinical profile.

### 3.3. Clinical Implications

The results of this randomized controlled trial carry several important clinical implications for the treatment of MD, particularly among men who engage in anabolic steroid and PED use. First, the observed reductions in MDDI scores—including improvements in the Drive for Size, Appearance Intolerance, and Functional Impairment—support the integration of manualized CBT protocols as a first-line intervention for individuals exhibiting the MD symptomatology. These findings reinforce the potential of CBT to address both the cognitive distortions and behavioral compulsions associated with the disorder, even in high-risk populations.

Second, the significant post-treatment changes in depressive symptoms (PHQ-9), psychological distress (K10), and disordered eating patterns (EDE-Q) indicate that MD often co-occurs with broader emotional and behavioral dysregulation. Clinicians should therefore adopt a transdiagnostic approach that targets comorbidities alongside body image-specific concerns. Screening for depression, exercise addiction, and eating pathology should be routine in settings that cater to fitness-focused male populations, such as gyms or sports medicine clinics.

Third, this study’s inclusion of participants with active steroid use suggests that psychological intervention remains effective even when biological or pharmacological risk factors are present. This challenges the common clinical assumption that individuals using PEDs are poor candidates for psychotherapy. Our findings suggest that motivational strategies within CBT, combined with body image restructuring, can produce meaningful therapeutic gains regardless of the substance use status.

Finally, the visual shifts in the body image perception observed through the Bodybuilder Image Grid (BIG) support the clinical value of incorporating perceptual tools in assessment and psychoeducation. Discussing clients’ selections on the BIG may provide a concrete, culturally sensitive way to externalize and challenge distorted muscular ideals. Such tools can enrich therapy by making abstract body image concerns more tangible and therapeutically actionable.

### 3.4. Limitations

Several limitations should be acknowledged when interpreting the findings of this study. First, the sample size, while adequately powered for detecting medium effect sizes, was relatively modest, potentially limiting the ability to detect smaller effects or to conduct more nuanced subgroup analyses. Second, the sample consisted exclusively of male participants recruited from a single country, which restricts the generalizability of the findings. Cultural norms surrounding masculinity, body image, and substance use may vary significantly across populations, and future studies should aim to include more diverse and gender-inclusive samples across multiple cultural contexts.

Third, this study did not incorporate a long-term follow-up period beyond the immediate post-intervention assessment. As a result, the durability of treatment effects—particularly in relation to relapse prevention, sustained cognitive restructuring, and the long-term cessation of AAS/PED use—remains unclear. Extended follow-up periods are necessary to determine whether the benefits of the intervention persist over time or require booster sessions. Lastly, although the intervention was delivered by trained clinicians under supervision, the potential influence of therapist effects and fidelity variability should be considered, despite adherence monitoring.

## 4. Methods

### 4.1. Study Design

This study was designed as a parallel-group, open-label randomized controlled trial (RCT) to investigate the clinical efficacy of cognitive behavioral therapy in reducing muscle dysmorphia symptoms and related psychological distress among males engaged in non-medical use of anabolic steroids or PEDs. This study was prospectively registered on ClinicalTrials.gov (Identifier: NCT06781853) and followed the CONSORT guidelines for the reporting of randomized trials.

Participants were randomly allocated to one of two arms: a CBT intervention group or a waitlist control group. Randomization was conducted using a computerized algorithm with a 1:1 allocation ratio after baseline eligibility screening. No stratification or blocking procedures were employed. This study employed repeated measures across three time points—baseline (T1), post-intervention (T2), and three-month follow-up (T3)—to evaluate both short- and medium-term treatment effects.

The trial was jointly conducted under the collaboration of three academic institutions: İstanbul Nişantaşı University, Beykoz University, and Üsküdar University. Data collection, participant recruitment, and therapy delivery were coordinated across these centers. Additionally, in specific cases requiring further psychiatric evaluation or diagnostic clarification, participants were referred to the outpatient psychiatry clinic of Prof. Dr. Gökben Hızlı Sayar (GHS), where clinical interviews were conducted via Microsoft Teams. All therapeutic interventions were carried out remotely using Microsoft Teams, allowing for standardized delivery and consistent therapist monitoring across sites.

### 4.2. G*Power Analysis

An a priori power analysis was conducted using G*Power 3.1 to determine the required sample size for detecting statistically significant group-by-time interaction effects. A repeated-measures ANOVA with two groups (CBT vs. control) and three time points (T1, T2, T3) was assumed. With an expected medium effect size (f = 0.25), alpha level set at 0.05, and statistical power (1–β) of 0.80, the minimum required sample size was calculated to be 54 participants. To mitigate the potential impact of attrition on statistical validity, the target sample size was set slightly higher, with 59 participants enrolled at baseline to ensure sufficient power for detecting interaction effects in the final analyses.

### 4.3. Inclusion and Exclusion Criteria

Participants were included in the study if they met the following eligibility criteria: (1) identified as male, (2) aged between 18 and 65 years, (3) engaged in regular gym attendance of at least three times per week, (4) reported a minimum of one year of non-medical use of anabolic steroids and/or PEDs, and (5) expressed willingness to undergo a structured psychiatric evaluation and participate in a 12-week CBT intervention with a three-month follow-up assessment.

Exclusion criteria were established to minimize confounding factors and ensure clinical safety. Individuals were excluded if they (1) met diagnostic criteria for severe psychiatric conditions such as major depressive disorder, bipolar disorder, substance use disorder (excluding steroids/PEDs), or antisocial personality disorder, (2) were currently prescribed or using psychotropic medications, (3) reported medical use of steroids or PEDs (e.g., for hormonal deficiencies or chronic illness), or (4) disclosed concurrent use of illicit substances such as cannabis, heroin, or other recreational drugs.

### 4.4. Participants and Recruitment

Participants were recruited through targeted, in-person outreach conducted at 21 fitness centers located across six districts of Istanbul: Kartal, Beykoz, Kadıköy, Sarıyer, Beyoğlu, and Gaziosmanpaşa. The recruitment team consisted of two master’s-level clinical psychology graduate students (NYY and EA) under the supervision of the lead author. These trained personnel approached potential participants directly outside gym facilities and conducted initial eligibility screenings based on behavioral and self-reported indicators of muscle dysmorphia and steroid/PED use.

A total of 71 individuals agreed to participate, provided written informed consent, and completed detailed sociodemographic questionnaires. Clinical interviews were conducted online by a senior psychiatrist (GHS) and assisted by the second author, senior psychologist (EY), employing DSM-5-TR criteria to assess body dysmorphic disorder with muscle dysmorphia specifier and non-medical steroid/PED use. Following this assessment, 63 individuals met full inclusion criteria and were enrolled in the study.

Of the 63 enrolled participants, 32 were randomly assigned to the intervention group and 31 to the control group. During the intervention phase, two participants in the intervention group dropped out—one due to family obligations and one due to hospitalization for an unrelated medical condition. In the control group, two participants discontinued: one lost contact, and one failed to complete the follow-up assessment. As a result, the final sample comprised 30 participants in the intervention group and 29 in the control group who completed all required assessments and were included in the final analyses.

### 4.5. Randomization Procedure

Participants who met eligibility criteria and completed baseline assessments were randomly assigned to either the intervention or control group using a computer-generated block randomization sequence with a 1:1 allocation ratio. Randomization was stratified by sex to ensure balanced distribution across groups. Allocation concealment was maintained using sealed, opaque, and sequentially numbered envelopes prepared by an independent researcher not involved in participant enrollment or assessment. Envelopes were opened by the study coordinator only after completion of all baseline measures to minimize selection bias.

### 4.6. Handling of Missing Data

All analyses were conducted on an intention-to-treat (ITT) basis, including all participants as randomized. Missing outcome data were addressed using multiple imputation with chained equations under the assumption that data were missing at random (MAR). The imputation model included baseline covariates and outcome variables to preserve the integrity of the dataset. To ensure robustness, sensitivity analyses were performed using both complete case analysis and last observation carried forward (LOCF) methods. Patterns of missingness were examined to detect any systematic bias, and missing data rates were reported by group and time point.

### 4.7. Measurement Tools

#### 4.7.1. Muscle Dysmorphic Disorder Inventory (MDDI)

MDDI is a 13-item self-report measure originally developed by Hildebrandt et al. (2004) [82] to assess core symptomatology of MD, a subtype of body dysmorphic disorder characterized by pathological preoccupation with muscularity. The MDDI comprises three subscales: Drive for Size (DFS), Appearance Intolerance (AI), and Functional Impairment (FI). Items are rated on a 5-point Likert scale ranging from 1 (“Never”) to 5 (“Always”), yielding a total score range of 13 to 65, with higher scores indicating more severe MD symptoms. The MDDI was used as a continuous dimensional measure to assess symptom severity, while formal DSM-5-TR diagnoses of muscle dysmorphia were established via structured clinical interviews. Prior meta-analytic evidence supports the validity and generalizability of the MDDI as a robust measure of MD symptomatology across diverse samples (Cooper et al., 2020 [64]). The Turkish version of the MDDI was validated by Devrim and Bilgiç (2019) [83] among 120 male bodybuilders. Exploratory factor analysis confirmed the original three-factor structure (DFS: 5 items, AI: 4 items, FI: 4 items), explaining 50.2% of total variance. Internal consistency was acceptable, with Cronbach’s alpha values of 0.73 (DFS), 0.66 (AI), 0.60 (FI), and 0.66 for the total score. Test–retest reliability over a two-week interval yielded an intraclass correlation coefficient (ICC) of 0.84 for the total MDDI. The Turkish adaptation demonstrated significant convergent validity with related constructs, such as the Eating Attitudes Test (EAT-40), body fat percentage, and the Fat-Free Mass Index (FFMI). Effect sizes (Cohen’s d) reported in prior intervention studies using MDDI typically range from 0.50 to 0.80 for reductions in MD symptoms following structured CBT programs, indicating medium to large treatment effects.

#### 4.7.2. Bodybuilder Image Grid (BIG)

The Bodybuilder Image Grid (BIG), also developed by Hildebrandt et al. (2004) [82], is a visual measure designed to assess perceptual and attitudinal dimensions of male body image, particularly in relation to muscularity and leanness. It consists of two formats: the BIG-Original (BIG-O), in which figures are numbered, and the BIG-Scaled (BIG-S), in which numeric labels are removed. Both formats present a matrix of 30 male silhouettes that systematically vary across Fat-Free Mass Index (FFMI) and body fat percentage. FFMI values increase from top to bottom (15.5 to 29.0 kg/m^2^), while body fat percentage increases from left to right (6.5% to 36%). The BIG-S includes four key items where participants identify the figure that best represents (1) their current body, (2) their ideal body, (3) the most attractive male body, and (4) the body they believe is most attractive to women. In the Turkish validation study by Devrim and Bilgiç (2019) [83], BIG-O and BIG-S demonstrated excellent test–retest reliability (ICC = 0.91–0.98) and significant convergent validity with MDDI scores, actual FFMI, and body fat percentage. The BIG-S was found to be more user-friendly and culturally comprehensible. Though the BIG is not intended for diagnostic purposes, it serves as a sensitive and visual tool to detect perceptual discrepancies and body image dissatisfaction among male populations with muscularity concerns.

#### 4.7.3. Kessler Psychological Distress Scale (K10)

K10 is a 10-item self-report instrument developed by Kessler et al. (1996) [84] to assess nonspecific psychological distress, primarily symptoms related to depression and anxiety. Respondents rate the frequency of each symptom over the past four weeks using a 5-point Likert scale ranging from 1 (“None of the time”) to 5 (“All of the time”), producing a total score between 10 and 50. Higher scores reflect greater levels of psychological distress, with clinical cut-offs typically suggested as follows: 10–15 (low), 16–21 (moderate), 22–29 (high), and 30–50 (very high distress). The scale has been widely used in epidemiological and clinical settings due to its brevity and sensitivity to distress across populations.

The Turkish adaptation and validation of the K10 was conducted by Altun (2019) [78] in a sample of university students. Confirmatory factor analysis supported a unidimensional structure, consistent with the original scale, and indicated good model fit (RMSEA = 0.06, CFI = 0.95, GFI = 0.94). Internal consistency was high (Cronbach’s alpha = 0.88), and test–retest reliability over a two-week interval yielded an ICC of 0.82. The Turkish version also demonstrated significant convergent validity with measures of depression (BDI) and anxiety (BAI). In intervention studies using the K10, Cohen’s *d* effect sizes typically range from 0.40 to 0.90 depending on the clinical severity of the sample and treatment type, reflecting moderate to large reductions in psychological distress following CBT or other psychotherapeutic interventions.

#### 4.7.4. Patient Health Questionnaire-9 (PHQ-9)

PHQ-9 is a widely used self-report scale developed by Kroenke and colleagues (2009) [85] for the screening, diagnosis, and monitoring of depressive symptoms in accordance with DSM-IV criteria. The scale consists of 9 items that assess the frequency of core depressive symptoms over the past two weeks. Each item is rated on a 4-point Likert scale from 0 (“Not at all”) to 3 (“Nearly every day”), yielding a total score range of 0 to 27. Severity thresholds are commonly interpreted as follows: 0–4 (minimal), 5–9 (mild), 10–14 (moderate), 15–19 (moderately severe), and 20–27 (severe depression).

The Turkish adaptation was validated by Güleç and colleagues (2012) [86] in a general adult population. The study confirmed the unidimensional structure of the PHQ-9 through confirmatory factor analysis, reporting satisfactory model fit indices (RMSEA = 0.063, CFI = 0.98, GFI = 0.97). The internal consistency of the Turkish version was high (Cronbach’s alpha = 0.86), and test–retest reliability over a two-week interval showed an intraclass correlation coefficient (ICC) of 0.83. Convergent validity was supported through significant correlations with the Beck Depression Inventory (BDI) and the General Health Questionnaire (GHQ-12). In clinical intervention trials, reductions in PHQ-9 scores typically correspond to medium-to-large effect sizes (Cohen’s *d* = 0.50–1.10) following CBT, pharmacotherapy, or combined treatments, reflecting strong sensitivity to change in depressive symptomatology.

#### 4.7.5. Eating Disorder Examination Questionnaire (EDE-Q)

The Eating Disorder Examination Questionnaire (EDE-Q) is a 28-item self-report instrument developed by Fairburn and Beglin (1994) [87] to assess the severity of eating disorder psychopathology across both cognitive and behavioral domains. It evaluates four subscales: Restraint, Eating Concern, Shape Concern, and Weight Concern. Each item is rated on a 7-point scale ranging from 0 (“No days” or “Not at all”) to 6 (“Every day” or “Markedly”), with higher scores indicating greater eating pathology. The global score is calculated as the mean of the four subscales. In addition to attitudinal items, the EDE-Q includes six behavioral frequency items addressing binge eating, vomiting, laxative misuse, and excessive exercise over the past 28 days.

The Turkish validation of the EDE-Q was conducted by Yücel and colleagues (2011) [88], who confirmed the four-factor structure through exploratory and confirmatory factor analyses. The internal consistency of the subscales ranged from 0.72 to 0.89, and the global scale yielded a Cronbach’s alpha of 0.90. Test–retest reliability over two weeks ranged from 0.81 to 0.91. Convergent validity was demonstrated through significant correlations with the Eating Attitudes Test (EAT-26) and the Beck Depression Inventory. The Turkish version was found to be a psychometrically sound tool for use in both clinical and non-clinical populations. Cohen’s *d* values reported in clinical trials targeting disordered eating symptoms typically range from 0.60 to 1.20, indicating large effect sizes in response to CBT-based interventions.

#### 4.7.6. Exercise Addiction Inventory (EAI)

The Exercise Addiction Inventory (EAI) is a brief self-report instrument developed by Terry, Szabo, and Griffiths (2004) [89] to identify individuals at risk of exercise addiction. It consists of 6 items rated on a 5-point Likert scale from 1 (“Strongly disagree”) to 5 (“Strongly agree”), yielding a total score range from 6 to 30. Higher scores indicate a greater risk of problematic exercise behavior. The EAI is based on the components model of addiction and evaluates salience, conflict, mood modification, tolerance, withdrawal symptoms, and relapse. A cut-off score of ≥24 is often used to classify individuals as “at risk” for exercise addiction, while scores of 13–23 suggest symptomatic use and ≤12 reflects asymptomatic levels.

The Turkish adaptation was conducted by Aydın and colleagues (2023) [90] in a sample of 548 physically active individuals. The confirmatory factor analysis supported the single-factor model with adequate model fit (CFI = 0.95, RMSEA = 0.071). The internal consistency of the scale was reported as α = 0.79, and test–retest reliability over a two-week interval was r = 0.87. Convergent validity was demonstrated through significant correlations with measures of obsessive exercise, body dissatisfaction, and compulsive behavior. The Turkish version preserved the clinical utility of the original scale and was recommended for both research and applied settings in populations engaged in regular physical activity. In intervention studies, reductions in EAI scores following CBT or psychoeducation typically yield Cohen’s *d* values ranging from 0.50 to 0.90, reflecting moderate to large effects.

### 4.8. Intervention

This CBT protocol was specifically tailored to address the unique psychological features of muscle dysmorphia in individuals with a history of steroid and PED misuse. The treatment emphasized restructuring maladaptive muscularity-related beliefs, reducing compulsive training and appearance-checking behaviors, and improving emotional regulation. Modules were adapted to target cognitive distortions amplified by substance use and body schema rigidity. Throughout therapy, participants engaged in body image exposure, behavioral experiments, and value-based goal setting to support long-term change.

Participants randomized to the experimental group received 12 manualized individual CBT sessions, each lasting approximately 50 min, delivered weekly over three months via Microsoft Teams. The intervention was specifically adapted for individuals exhibiting symptoms of muscle dysmorphia and non-medical steroid or PEDs use. The protocol targeted maladaptive beliefs about muscularity, perfectionism, and self-worth, with additional modules on cognitive restructuring of body-related distortions, behavioral experiments to reduce compulsive checking and reassurance-seeking, and graded exposure to avoided situations such as gym absences or wearing looser clothing. Homework assignments included thought logs, mirror exposure, and behavioral activation tasks addressing over-exercise patterns.

Sessions 1–2 focused on psychoeducation and functional analysis of body image behaviors; sessions 3–6 addressed cognitive distortions and body dissatisfaction; sessions 7–9 incorporated behavioral modification techniques to reduce compulsive training, steroid rituals, and food restriction; and sessions 10–12 emphasized relapse prevention, identity reconstruction, and value-based goal setting. Fidelity to the treatment model was supported by therapist feedback and weekly supervision. Participants were required to attend a minimum of 9 out of 12 sessions (75%) to be included in the per-protocol analysis.

The control group received no therapeutic intervention during the study period but completed all assessments. Participants in the control condition were offered access to the CBT program following the study’s conclusion if they met the same eligibility criteria at that time (Please See Supplementary Materials for Full CBT Protocol).

### 4.9. Procedure

After obtaining written informed consent, participants completed a battery of baseline assessments including clinical interviews, sociodemographic forms, and psychometric scales. Eligible participants were then randomly assigned to either the intervention group (n = 30) or the control group (n = 29) using a computerized random number generator to ensure allocation concealment. Randomization was conducted by an independent researcher who was not involved in assessment or therapy delivery.

Participants in the intervention group received 12 consecutive weekly CBT sessions via Microsoft Teams, while the control group received no active treatment during the study period. Outcome assessments were conducted at three time points: baseline (T1), immediately post-treatment (T2; approximately 12 weeks after randomization), and three-month follow-up (T3). All self-report instruments were administered electronically via secure links and completed independently by the participants.

Adherence to the intervention was monitored through therapist session logs. Participants in the intervention arm were required to attend at least 9 out of 12 sessions (≥75%) to be included in the per-protocol analysis. Attendance, dropout reasons, and session completion rates were tracked throughout the study. Dropouts and missing follow-up data were handled according to the intention-to-treat principle, with last observation carried forward (LOCF) imputation where necessary. No adverse events or harm were reported during the study period.

### 4.10. Therapists and Quality Assurance

CBT sessions were delivered by three authors (MÇ, EY, SVÜ), each of whom holds an assistant professorship in clinical/applied psychology and has undergone formal certification in CBT. All therapists possessed prior clinical experience in treating individuals with body image disturbances, MD, and substance-related disorders. To ensure treatment fidelity and maintain therapeutic consistency across therapists, weekly group supervision sessions were conducted by a senior professor of psychiatry (GHS), who also oversaw the clinical screening process for inclusion.

To monitor adherence to the CBT protocol, a fidelity monitoring committee was formed, comprising two senior CBT clinicians (independent of the research team), a faculty member with a PhD in clinical psychology, and one psychiatrist. The committee reviewed a stratified random sample of three 15 min video segments from each therapist, covering early (sessions 1–3), mid (sessions 6–7), and late (sessions 10–12) phases of treatment. Fidelity assessments focused on adherence to core CBT principles, intervention-specific modules, therapist competence, and ethical conduct. Feedback was provided in a structured format, and all therapists were deemed adherent to the manualized protocol. No serious protocol violations or ethical concerns were reported throughout the study.

All therapists participated in preparatory meetings prior to treatment delivery to ensure a shared understanding of the manualized protocol. These sessions involved structured case discussions, protocol walkthroughs, and rehearsal of intervention-specific techniques tailored to individuals with muscle dysmorphia and PED misuse. The training emphasized consistency in cognitive restructuring, behavioral strategies, and managing high-risk clinical presentations. Throughout the intervention period, therapists engaged in reflective discussions during weekly supervision with the senior psychiatrist (GHS), allowing for real-time consultation on case complexities, ethical dilemmas, and therapeutic decision-making. This integrated model of pre-treatment preparation and ongoing expert supervision ensured high fidelity and clinical responsiveness across treatment delivery (Please See Supplementary Materials for Adherence and Quality Assurance Report).

### 4.11. Statistical Analyses

All statistical analyses were performed using IBM SPSS Statistics (Version 29.0) and Python (Version 3.13.3) with relevant statistical libraries. Descriptive statistics were computed to summarize demographic and baseline characteristics across groups. The primary analytic approach involved repeated-measures analysis of variance (RM-ANOVA) to evaluate time × group interactions on outcome variables across three time points: baseline (T1), post-intervention (T2), and three-month follow-up (T3).

Prior to conducting RM-ANOVA, data were screened for normality, outliers, and violations of sphericity. When Mauchly’s test indicated a violation of the sphericity assumption, Greenhouse–Geisser corrections were applied. Between-group comparisons at each time point were supplemented with independent samples *t*-tests, and within-group changes over time were examined using paired samples *t*-tests where appropriate.

Effect sizes were calculated and reported using partial eta squared (η^2^) for ANOVA analyses and Cohen’s *d* for pairwise comparisons, with conventional benchmarks of 0.01 (small), 0.06 (medium), and 0.14 (large) for η^2^ and 0.20 (small), 0.50 (medium), and 0.80 (large) for *d*. All tests were two-tailed with α set at 0.05.

The primary analyses adhered to the intention-to-treat (ITT) principle. Missing outcome data due to dropout or incomplete assessments were addressed using the last observation carried forward (LOCF) method. Sensitivity analyses were conducted to compare ITT and per-protocol results; no significant differences were observed. All analyses were independently verified by a statistician blinded to group allocation.

### 4.12. Ethical Considerations

This study was approved by the Üsküdar University Non-Invasive Clinical Research Ethics Committee (Approval No: 61351342/020-319) and was conducted in accordance with the ethical standards outlined in the Declaration of Helsinki. All participants provided written informed consent prior to enrollment, including explicit consent for the use of anonymized data in academic publications. Participants were informed of their right to withdraw from the study at any time without prejudice or loss of benefits.

Confidentiality was ensured through secure digital storage of all data, with personal identifiers removed prior to analysis. Electronic data were encrypted, and only authorized research personnel had access. Video recordings of therapy sessions used for fidelity monitoring were stored on password-protected institutional servers and deleted following review.

Participants assigned to the control group were offered access to the same CBT program in an accelerated format upon completion of the study assessments. No adverse events or ethical breaches were reported during the course of the study.

## 5. Conclusions

This randomized controlled trial offers preliminary but compelling evidence that a structured CBT protocol can effectively reduce MD symptoms and comorbid psychopathology in male individuals engaged in the non-medical use of AASs and other PEDs. The observed improvements across MD-specific domains, mood disturbances, psychological distress, disordered eating, and compulsive exercise behaviors underscore the multidimensional nature of MD and the transdiagnostic reach of CBT in this high-risk clinical subgroup.

By demonstrating therapeutic efficacy in a population often considered refractory due to concurrent substance misuse, this study contributes to a critical and underdeveloped area in the treatment literature. The findings suggest that targeted psychological interventions can address both the cognitive–behavioral and affective sequelae associated with the pharmacologically driven body image pathology. Future research should evaluate the long-term maintenance of treatment gains, explore the integration of adjunctive strategies such as motivational enhancement or relapse prevention modules, and expand generalizability through more diverse samples and settings.

## Figures and Tables

**Table 1:** Baseline characteristics of participants in the CBT (Cognitive Behavioral Therapy) and control groups. Measures: MDDI (Muscle Dysmorphic Disorder Inventory), PHQ-9 (Patient Health Questionnaire–9), K10 (Kessler Psychological Distress Scale), BIG (Bodybuilder Image Grid), EDE-Q (Eating Disorder Examination Questionnaire), and EAI (Exercise Addiction Inventory).

Variable	CBT Group (n = 30)	Control Group (n = 29)	*p*-Value
Age, mean (SD)	27.8 (6.2)	28.5 (5.9)	0.618
Education, years, mean (SD)	14.1 (2.4)	14.5 (2.7)	0.522
MDDI Total, mean (SD)	42.3 (6.8)	41.7 (7.1)	0.701
PHQ-9 Total, mean (SD)	11.6 (4.5)	12.1 (4.2)	0.645
K10 Total, mean (SD)	28.4 (6.2)	29.0 (5.7)	0.698
BIG Ideal–Current Discrepancy	2.4 (1.1)	2.5 (1.0)	0.738
EDE-Q Global, mean (SD)	3.1 (1.0)	3.0 (1.2)	0.814
EAI Total, mean (SD)	20.7 (3.5)	21.0 (3.7)	0.752

**Table 2:** Changes in MDDI (Muscle Dysmorphic Disorder Inventory) scores across time points for CBT and control groups.

Time Point	CBT Group (n = 30) Mean (SD)	Control Group (n = 29) Mean (SD)	F (1, 57)	*p*-Value	η^2^ Partial
Baseline	42.3 (6.8)	41.7 (7.1)			
Post-Treatment	34.9 (6.1)	40.9 (6.9)	15.72	<0.001	0.216
3-Month Follow-Up	35.2 (6.4)	40.5 (7.0)	13.94	0.001	0.197

**Table 3:** Changes in secondary outcome measures across time points (T1 = Baseline, T2 = Post-Treatment, T3 = 3-Month Follow-Up). CBT: Cognitive Behavioral Therapy. Measures: PHQ-9, K10, EDE-Q, EAI, and BIG.

Measure	Group	T1 Mean (SD)	T2 Mean (SD)	T3 Mean (SD)
PHQ-9	CBT (n = 30)	14.1 (4.2)	8.3 (3.7)	7.1 (3.9)
	Control (n = 29)	13.8 (4.5)	13.2 (4.6)	12.6 (4.3)
K10	CBT	28.4 (6.5)	21.1 (5.8)	18.7 (5.2)
	Control	27.9 (6.3)	27.4 (6.0)	26.9 (5.8)
EDE-Q (Global Score)	CBT	3.42 (0.88)	2.26 (0.93)	1.91 (0.95)
	Control	3.38 (0.85)	3.31 (0.87)	3.24 (0.90)
EAI	CBT	23.2 (3.9)	18.1 (4.2)	16.4 (4.6)
	Control	22.9 (4.1)	22.3 (4.3)	21.7 (4.0)
BIG Discrepancy Score	CBT	4.1 (1.3)	2.4 (1.1)	1.9 (1.2)
	Control	4.0 (1.4)	3.8 (1.3)	3.6 (1.2)

**Table 4:** Repeated-measures ANOVA results for group × time effects. Measures: MDDI (Muscle Dysmorphic Disorder Inventory), PHQ-9 (Patient Health Questionnaire–9), K10 (Kessler Psychological Distress Scale), EDE-Q (Eating Disorder Examination Questionnaire), EAI (Exercise Addiction Inventory), and BIG (Bodybuilder Image Grid).

Outcome Measure	F (Group × Time)	*p*-Value	Partial η^2^	Interpretation
MDDI (Total)	22.17	<0.001	0.28	Large effect
PHQ-9	16.35	<0.001	0.23	Large effect
K10	14.02	<0.001	0.21	Large effect
EDE-Q (Global)	10.94	0.001	0.18	Medium effect
EAI	12.63	<0.001	0.20	Large effect
BIG Discrepancy	9.01	0.004	0.16	Medium effect

**Table 5:** Paired sample *t*-tests for pre- to post-treatment changes in the CBT group. Measures: BIG (Bodybuilder Image Grid), K10 (Kessler Psychological Distress Scale), PHQ-9 (Patient Health Questionnaire–9), EDE-Q (Eating Disorder Examination Questionnaire), and EAI (Exercise Addiction Inventory).

Measure	Pre-Treatment Mean (SD)	Post-Treatment Mean (SD)	t (df)	*p*-Value	Cohen’s d
BIG	17.6 (2.5)	15.8 (2.4)	4.32 (29)	<0.001	0.79
K10	29.2 (4.3)	24.6 (3.9)	5.10 (29)	<0.001	0.93
PHQ-9	14.5 (3.8)	10.8 (3.5)	5.36 (29)	<0.001	0.98
EDE-Q	3.2 (0.7)	2.6 (0.6)	4.05 (29)	<0.001	0.74
EAI	23.4 (3.1)	19.9 (2.8)	5.88 (29)	<0.001	1.07

## Data Availability

Data is contained in the paper.

## Supplementary Materials

Supplementary Materials for this study are available at the following Figshare repository: https://doi.org/10.6084/m9.figshare.29366687. These materials include three core documents. First, the Full CBT Protocol outlines the complete 12-session manualized cognitive behavioral therapy program specifically adapted for muscle dysmorphia in male steroid users. It includes the structured goals, session content, techniques, and weekly homework plans, offering a replicable intervention framework. Second, the Adherence and Quality Assurance Report provides a summary of therapist fidelity monitoring procedures, detailing adherence ratings, competence levels, ethical compliance, and reviewer comments across early, mid, and late therapy phases. This document confirms the consistent and high-quality implementation of the intervention. Third, the Therapeutic Narratives file contains anonymized session excerpts illustrating key clinical shifts such as cognitive restructuring, mirror exposure, gym avoidance, identity work, and relapse prevention. These narratives reflect the depth of participant engagement and provide a qualitative context to the observed quantitative outcomes.

## Abbreviations

The following abbreviations are used in this manuscript:

MD	Muscle Dysmorphia
AAS	Anabolic–Androgenic Steroids
PEDs	Performance-Enhancing Drugs
CBT	Cognitive Behavioral Therapy
BDI-II	Beck Depression Inventory-II
BAI	Beck Anxiety Inventory
EDE-Q	Eating Disorder Examination Questionnaire
EAI	Exercise Addiction Inventory
MDDI	Muscle Dysmorphic Disorder Inventory
BIG	Bodybuilder Image Grid
